# Successful endoscopic removal of a rare, large impacted pancreatic duct stone using grasping forceps: A case report with video

**DOI:** 10.1097/MD.0000000000010304

**Published:** 2018-04-06

**Authors:** Qin Liu, Yiping Wang, Hongze Zeng, Bing Hu

**Affiliations:** a Department of Gastroenterology, West China Hospital, Sichuan University, Chengdu; b Department of Gastroenterology, Affiliated Cixi People's Hospital, Wenzhou Medical University, Ningbo, Zhejiang, China.

**Keywords:** endoscopic lithoextraction, endoscopic retrograde cholangiopancreatography, pancreatic duct stones, pancreatolithiasis

## Abstract

**Rationale:**

As a main complication of chronic pancreatitis (CP), pancreatic duct stones (PDSs) are often associated with ductal obstruction resulting in increasing intraductal and parenchymal pressure and long-lasting pain. There are many methods for removing PDSs. However, for large stones, it is technically difficult to remove them entirely by endoscopic retrograde cholangiopancreatography (ERCP).

**Patient concerns:**

A 57-year-old man presented with a chief complaint of severe epigastric pain radiating to his back 3 or 4 times annually for 2 years.

**Diagnosis:**

The abdominal computed tomography scan revealed dilation of the proximal pancreatic duct with an irregular high-density calcification shadow located at the head of the pancreas.

**Interventions:**

A pancreatic stent was placed initially by ERCP to relieve epigastric pain and alleviate symptom. Repeated ERCP was subsequently performed 2 months later to extract the impacted large pancreatic stone.

**Outcome:**

By using grasping forceps, the huge coralloid stone (272 × 0 mm) was successfully extracted in an en bloc manner. Then, a 7-French × 5 cm plastic pancreatic stent was placed for 2 weeks. The patient was free of any pancreatic pain during the 2-year follow-up.

**Lessons:**

Generally, for large or impacted pancreatic stones, endoscopic removal is technically difficult to achieve. Pancreatic stenting can be an effective method of alleviating abdominal pain and facilitating subsequent endoscopic lithoextraction. Extraction of large stones with grasping forceps can be an alternative approach instead of extracorporeal shock wave lithotripsy or surgery when stones are impacted at the papilla's orifice and partially protruding into the duodenal lumen.

## Abstract 2

Supplemental Digital Content is available in the text

## 1 Introduction

Pancreatic duct stones (PDSs) are pathognomonic signs of chronic pancreatitis (CP), and about 90% of patients have longstanding CP combined with PDSs.^[1]^ The natural course of CP is usually complicated by stones within the main pancreatic duct (MPD).^[2]^ Stones or calculus may lead to obstruction in pancreatic ducts and hypertension in the pancreas; therefore, they can cause relapsed pancreatitis and intense epigastric pain.^[3]^ For CP, it is crucial to relieve patients’ pain and alleviate their symptom by performing MPD drainage with removal of the obstructing PDSs.^[4]^

Compared with operation, microinvasive techniques for stone removal are preferred.^[4,5]^ Generally, smaller calculi located in the MPD can be extracted endoscopically without difficulty,^[6]^ whereas larger or impacted ones need extracorporeal shock wave lithotripsy (ESWL) before endoscopic retrograde cholangiopancreatography (ERCP).^[7]^ In this case, we describe successful endoscopic extraction of a rare, large impacted PDS using grasping forceps.

## 2 Case report

Our case report includes a retrospective and descriptive analysis, and informed consent was obtained from the patient.

**Figure 1:**
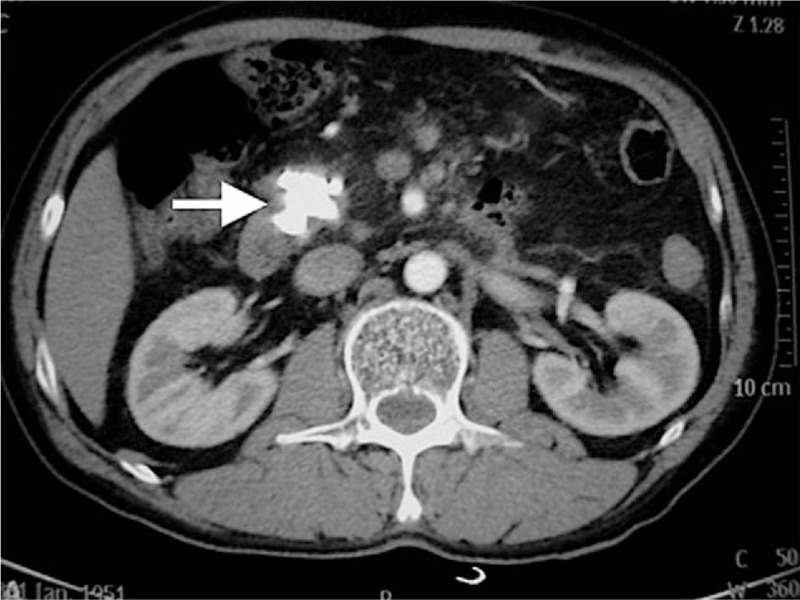
Abdominal computed tomography scan demonstrating a large high-density shadow located at the head of the pancreas (white arrow).

A 57-year-old man was admitted to our hospital for acute onset of chronic pancreatitis. He had experienced severe epigastric pain radiating to his back at least 4 times annually in the last 2 years without compliance of fever or diarrhea, and he had no history of diabetes. Blood test results showed an extremely high serum amylase level (2365 IU/L, normal range 30–110 IU/L). An abdominal computed tomography (CT) scan revealed atrophy of the pancreatic body and an irregular high-density calcification shadow (252 × 3 mm) in the head of the pancreas with dilation of the distal part of the MPD at 8 mm (Fig. 1). Accordingly, emergency ERCP was performed immediately. However, due to the large size of the stone and its close impaction with the narrow downstream duct, endoscopic removal was not attempted. A 5-French (Fr) × 10 cm pancreatic duct stent (Cook) was placed for drainage and pain relief.

**Figure 2:**
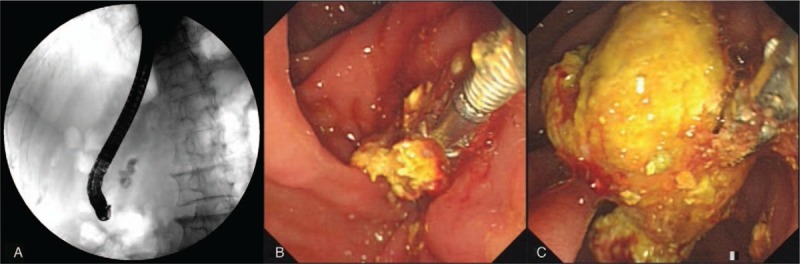
(A) Fluoroscopy demonstrating the huge radiopaque pancreatic stone under endoscopic retrograde cholangiopancreatography. (B, C) Endoscopic lithoextraction. A huge pancreatic stone is found impacted in the main pancreatic duct and partially protruding out of the papillary orifice. The stone is removed completely by grasping the protruded part of the stone using forceps.

**Figure 3:**
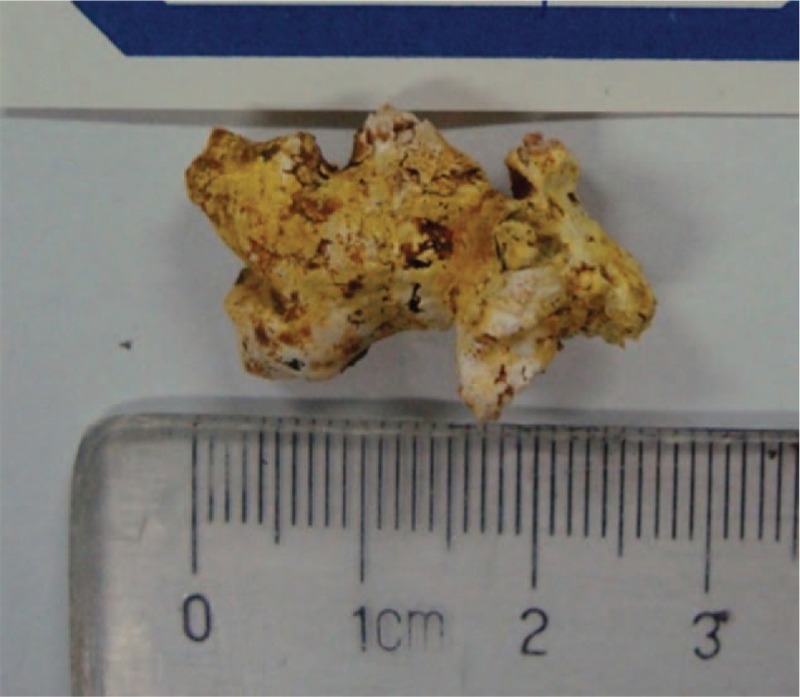
View of the removed pancreatic stone. The coral-like stone measures 272 × 0 mm.

Two months later, the blood test result showed that the serum amylase level decreased to 111 IU/L. Repeat ERCP was performed to remove the stent before conducting ESWL. The duodenoscope successfully reached the descending part of the duodenum, but surprisingly, part of the whitish-yellow impacted pancreatic stone was found extending into the duodenal lumen through the slightly swollen major papilla. With a good visual field under the duodenoscope, we were able to fully observe the stone and the orifice, and we decided to try to extract the stone under ERCP using grasping forceps. After endoscopic retrieval of the former stent, selective cannulation of the pancreatic duct was successfully attempted by using a sphincterotome, and then a small amount of contrast was slowly injected. Pancreatography demonstrated a huge filling defect (252 × 2 mm) associated with dilatation of the MPD to 6 mm. By using the grasping forceps (Rat Tooth; Olympus, Japan), the protruding part of the stone was firmly grasped, and we gradually extracted the huge coralliform stone (272 × 0 mm) in an en-bloc manner (Figures 2A–C, 3; see Video, Supplemental Video which demonstrates removal of the stone). To ensure fluent drainage of the MPD, another 7-Fr × 10 cm plastic stent was placed for 2 weeks. Consequently, the abdominal pain was obviously relieved.

The patient was free of symptoms such as abdominal pain during the 2-year follow-up.

## 3 Discussion

Pancreatolithiasis can be considered to represent a manifestation of advanced chronic pancreatitis.^[8]^ Pancreatolithiasis or PDSs often make the natural course of CP complicated, especially when stones are located in the MPD.^[2]^ A calculus in the pancreatic duct usually leads to obstruction, thus resulting in ductal and parenchymal hypertension of the pancreas that consequently causes severe pain. The mechanism of pain is multifactorial involving combinations of factors, and pancreatic ductal hypertension can be the primary contributor.^[9]^ Persistent epigastric pain is the main symptom of PDSs,^[10]^ and it often leads to use of analgesic drugs, loss of weight, inability to work, and reduced quality of life, and it can induce other complications, such as diabetes, steatorrhea, and jaundice. In addition, PDSs are significantly related to pancreatic carcinoma. Those with pancreatolithiasis are at an approximately 27-fold higher risk of developing pancreatic cancer than healthy individuals.^[11]^ Calculi clearance should be performed immediately once it is diagnosed.

Removing stones, alleviating obstruction, and decreasing ductal hypertension are principles of operation. Selection of the appropriate treatment method for pancreatolithiasis depends on the location, size, and number of stones.^[11,12]^ Operative treatment, ESWL, and endoscopic lithoextraction are main treatments.^[12]^ Generally, operation is recommended for diffusing PDSs filling ducts and stones exiting the tail of the pancreas.^[8]^ As for stones in MPD, operation is seldom the first-step treatment considering its invasiveness and associated morbidity. Extraction balloons and baskets can remove the stones by retrograde access of the pancreatic duct with minor invasion, but these methods do have certain limitations. Baskets can become trapped in the duct, and the stone or balloon may rupture. Large stones are usually treated by ESWL.^[7]^ In a recent multicenter study, ESWL was recommended as the first-line treatment of large stones (≥10 mm), and ERCP was recommended for stones less than 10 mm.^[12]^ However, complete stone clearance is difficult to achieve with ESWL when the PDSs are much larger than 10 mm. In fact, the aim of treatment for PDSs is to maintain patency of the MPD and relieve pain; thus, endoscopic treatment such as MPD stenting is still a good option whether the PDSs can be completely removed.

Endoscopic treatment of pancreatic stones is indicated in patients with chronic pancreatitis and stones in the main or accessory pancreatic duct, and especially those with complaints of abdominal pain. Smaller calculi within the MPD usually can be extracted endoscopically, but it is often impossible for large or impacted stones that require combined ESWL because of the toughness of the stones and the narrow space of the MPD lumen.^[6,9]^ However, there are still several cases of successful extraction of large pancreatic stones under ERCP,^[13–15]^ but the stones were much smaller than the stone reported in our case. In clinical practice, endoscopic treatment is often undertaken as adjunctive therapy after fragmentation of large pancreatic stones by ESWL. In the present case, the emergency ERCP we performed was to promote the recovery of acute onset of CP by pancreatic stenting rather than treating the patient for the PDS immediately. When the patient was in a stable condition, the stent was removed in order to minimize the possible post-ESWL complications, such as bleeding, pancreatitis, or perforation. Usually, stones larger than 10 mm in diameter or stone impaction is unfavorable for endoscopic removal, so ESWL or ESWL combined with endoscopic removal might be preferred for these cases.^[16]^ However, these procedures also add the cost of treatments and burden to patients without improving the outcome of pancreatic pain.^[17]^ Therefore, we tried to extract the impacted stone simultaneously when the stent was removed, and consequently, we succeeded. Endoscopic extraction of large pancreatic stones using grasping forceps is not a conventional method, but it can be attempted in cases for the good location and horizon of the stone, especially when the stones are near the orifice of the papilla.

## 4 Conclusion

We described the first case of such a huge impacted pancreatic stone successfully extracted by grasping forceps. Lithoextraction of large pancreatic stones under ERCP can be considered as a reasonable supplement to ESWL. Complete endoscopic clearance of impacted pancreatic stones leads to MPD patency and immediate pain relief. Large stones located in the head of the pancreas, especially when stones are impacted at the orifice of the papilla and partially protruding into duodenal lumen, can be candidates for using this straightforward approach to obtain a satisfactory therapeutic effect.

## Author contributions

**Supervision:** B. Hu.

**Writing – original draft:** Q. Liu.

**Writing – review & editing:** B. Hu, H. Zeng, Y. Wang.

## Supplementary Material

Supplemental Digital Content
